# Development of 3D Thermoplastic Polyurethane (TPU)/Maghemite (ϒ-Fe_2_O_3_) Using Ultra-Hard and Tough (UHT) Bio-Resin for Soft Tissue Engineering

**DOI:** 10.3390/polym14132561

**Published:** 2022-06-23

**Authors:** Ehsan Fallahiarezoudar, Nor Hasrul Akhmal Ngadiman, Noordin Mohd Yusof, Ani Idris, Mohamad Shaiful Ashrul Ishak

**Affiliations:** 1 Department of Industrial Engineering, Faculty of Engineering, East of Guilan, University of Guilan, Roudsar 44918, Guilan, Iran; fallahi.ehsan@guilan.ac.ir; 2 School of Mechanical Engineering, Faculty of Engineering, Universiti Teknologi Malaysia, Johor Bahru 81310, Johor, Malaysia; noordin@utm.my; 3 School of Chemical Engineering, Faculty of Engineering, c/o Institute of Bioproduct Development, Universiti Teknologi Malaysia, Johor Bahru 81310, Johor, Malaysia; aniidris@utm.my; 4 Faculty of Mechanical Engineering Technology, Universiti Malaysia Perlis, Kampus Pauh Putra, Arau 02600, Perlis, Malaysia; mshaiful@unimap.edu.my

**Keywords:** soft tissue engineering, bone scaffold, DLP 3D printing, mechanical strength, biocompatibility

## Abstract

The use of soft tissue engineering scaffolds is an advanced approach to repairing damaged soft tissue. To ensure the success of this technique, proper mechanical and biocompatibility properties must be taken into consideration. In this study, a three-dimensional (3D) scaffold was developed using digital light processing (DLP) and ultra-hard and tough (UHT) bio-resin. The 3D scaffold structure consisted of thermoplastic polyurethane (TPU) and maghemite (*ϒ*-Fe_2_O_3_) nanoparticles mixed with UHT bio-resin. The solution sample for fabricating the scaffolds was varied with the concentration of the TPU (10, 12.5, and 15% wt/v) and the amount of *ϒ*-Fe_2_O_3_ (1, 3, and 5% *v*/*v*) added to 15% wt/v of TPU. Before developing the real geometry of the sample, a pre-run of the DLP 3D printing process was done to determine the optimum curing time of the structure to be perfectly cured, which resulted in 30 s of curing time. Then, this study proceeded with a tensile test to determine the mechanical properties of the developed structure in terms of elasticity. It was found that the highest Young’s Modulus of the scaffold was obtained with 15% wt/v TPU/UHT with 1% *ϒ*-Fe_2_O_3_. Furthermore, for the biocompatibility study, the degradation rate of the scaffold containing TPU/UHT was found to be higher compared to the TPU/UHT containing ϒ-Fe_2_O_3_ particles. However, the MTT assay results revealed that the existence of ϒ-Fe_2_O_3_ in the scaffold improved the proliferation rate of the cells.

## 1. Introduction

In the human body, soft tissue refers to the tissue that connects, supports, and surrounds other structures. It includes muscles, tendons, ligaments, fascia, nerves fibrous tissue, fat, blood vessels, and synovial membranes. Regularly, soft tissue damage is caused by disease, congenital defects, trauma, and aging, which lead to the inability of the tissue to self-heal [1]. Due to this matter, an alternative technology called tissue engineering was proposed to help the healing process by regenerating or replacing the damaged tissue [2]. The important part of this method is the development of the scaffold, which is able to restore, maintain, and improve the function of tissue [3]. A scaffold is a template for tissue formation that allows the cells to migrate, adhere to, and produce tissue [4]. According to Li et al. [5], in the development of a successful and well-functioning scaffold, the following properties should be taken into account: (i) the biocompatibility and biodegradability of the scaffold in order to match the cell or tissue growth in vitro/vivo; (ii) suitable mechanical properties to match the tissue at the implantation site; (iii) proper surface chemistry for cell attachment, proliferation, and differentiation activity. In addition, the architecture of the scaffold in terms of porosity is also necessary to consider as it is important for cell growth, nutrient transportation, and metabolic waste [6,7]. In applying the tissue engineering method, the scaffold must satisfy these properties.

**Figure 1:**
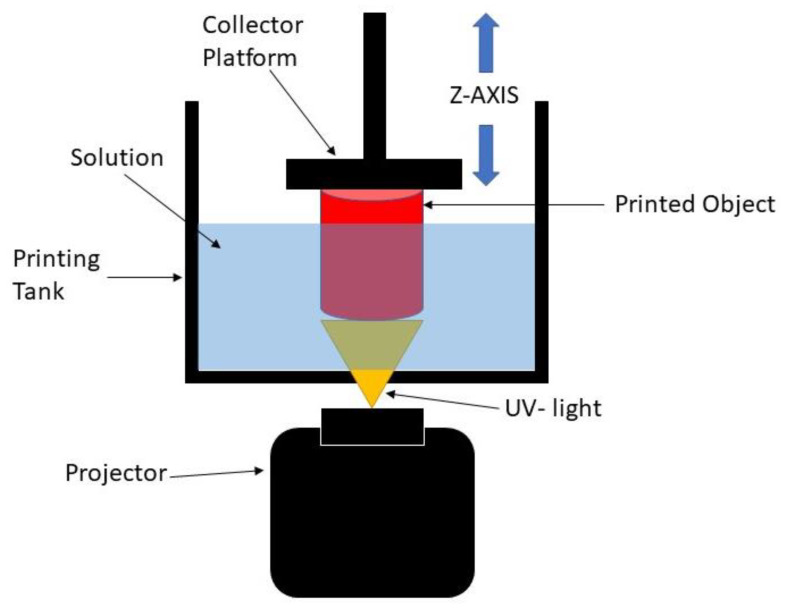
Schematic diagram of DLP 3D printing process.

The digital light processing (DLP) 3D printing technique is one of the best-defined techniques to produce scaffolds for soft tissue engineering. DLP is based on the basic principle of stereolithography (SLA), which offers better resolution and is more versatile than other conventional and additive manufacturing methods [8]. Practically, the DLP 3D printing process uses ultraviolet (UV) light to project the entire X and Y cross-sectional layers of the structure to be produced at one time onto a photopolymer resin, which will change the area exposed to UV light from a liquid to a solid. The solidified layer is formed on the collector, which is the Z axis, as shown in Figure 1. By using this process, the production time is reduced, which leads to higher productivity and reproducibility of the scaffold [9,10,11]. It is also able to create complex structures with highly accurate internal architecture as it has a high feature resolution [6]. Thus, this process can be used to develop the 3D structure of scaffolds with any kind of shape while maintaining good mechanical strength, and it can create a good environment for enhancing the biocompatibility performance [5]. However, it is worth noting that this method lacks resin as the resin must be capable of a photopolymerization reaction [12]. Therefore, UHT resin is used with the DLP 3D printer system. This resin is one of the standard bio-resins that can solidify quickly in the presence of a specific light source [11].

In recent years, researchers have been exploring the use of nanofiber-based scaffolding systems that can act as a scaffold for tissue engineering applications. This is because the structures produced by nanofiber scaffolds mimic the structure of natural human tissue and, thus, enhance the cell growth rate [13]. Considering this, efforts in finding the best materials and techniques for developing tissue engineering scaffolds that fulfill the requirements are still ongoing. In this study, biocompatible thermoplastic polyurethane (TPU) was used as it has been widely used in medical applications. This chosen material has important characteristics required for building bone scaffold, which represents good biocompatibility and flexibility compared to other types of synthesized polymers. Thermoplastic polyurethane (TPU) is a class of PU with excellent elastic and tear-resistant properties and moderate tensile strength. In our previous studies [14,15,16], TPU has been used as a novel mixture for fabricating engineered tissue for an aortic heart valve using the electrospinning process. As a continuation, this study focused on the adjustment of parameter settings for printing TPU using a DLP 3D printer before applying it to the various applications of soft tissue engineering scaffolds. Ultra-hard and tough (UHT) bio-resin acted as a curing agent to print the TPU. However, the use of the polymer itself with the resin did not meet the mechanical properties required of the scaffold [17].

Magnetic nanoparticles in the form of maghemite (γ-Fe_2_O_3_) have been used in biomedical applications [18,19,20,21,22], such as cell sheet construction, cell expansion, magnetic cell seeding, cancer hyperthermia treatment, and drug delivery. Maghemite (*ϒ*-Fe_2_O_3_) nanoparticles have been proven to enhance the mechanical properties of tissue engineering scaffolds. Maghemite (*ϒ*-Fe_2_O_3_) nanoparticles were chosen because of their low toxicity and ability to act as a reinforcing agent to the scaffold due to a larger surface area provided by the very fine nanoparticles [23,24,25,26,27,28]. In our previous work [29], the addition of maghemite (γ-Fe_2_O_3_) nanoparticles exhibited good biocompatibility. The presence of magnetic nanoparticles within scaffolds has also increased their rigidity favorably [30,31]. The material characterization of maghemite (γ-Fe_2_O_3_) nanoparticles has also been discussed in our previous works [10,11,14,15,16,17,32,33,34,35]. However, an excessive amount of maghemite used led to a decrease in the mechanical properties as the stiffness of the scaffold increased. *ϒ*-Fe_2_O_3_ nanoparticles also offer better cell adhesion properties and enhance the cell growth rate [11,36,37].

In addition, in a previous study by Fallahiarezoudar et al. [16], for the development of cardiac tissue engineering scaffold focusing on the aortic portion and using the electrospinning process, they established materials consisting of thermoplastic polyurethane (TPU) and maghemite (ϒ-Fe_2_O_3_) nanoparticles, and both of the materials showed good mechanical properties. However, a limitation occurred because of the process that they used, which was electrospinning. This process can only develop two-dimensional structures, and therefore, there is a limit to the strength of the scaffolds produced. Cardiac tissue engineering scaffolds need to be produced with 3D printing in order to obtain the appropriate mechanical and biocompatibility properties as well as to be implemented clinically.

In this study, a 3D structure of scaffold that consisted of TPU containing ϒ-Fe_2_O_3_ nanoparticles mixed with ultra-hard and tough (UHT) bio-resin was developed using the DLP 3D printing process. The aim of this study was to evaluate the performance of the 3D structure in terms of the mechanical and biocompatibility properties.

## 2. Materials and Methods

Almost all the chemicals used in this research were of analytical purity and no further purification was applied. The base material used for developing the 3D structure in this study was thermoplastic polyurethane (TPU). Flexible elastomer TPU granules with Shore A 60 hardness, an Mw of 90 kDa, and a glass transition temperature of −50 °C were purchased from Wenzhou City Sanho Co., Ltd. Maghemite (ϒ-Fe_2_O_3_) was used as a filler to enhance the properties of the 3D structure, while ultra-hard and tough (UHT) bio-resin acted as a resin that bound the TPU to the maghemite. UHT also reacted with the ultraviolet (UV) light to form the 3D structure. The chemicals used in this study were reagent grade and included the following: iron (II) chloride (FeCl2) (98% purity, Sigma Aldrich, St. Louis, MO, USA), iron (III) chloride (FeCl3) (45% purity, Riedel-de Haen), sulfuric acid (H_2_SO_4_) (QRëC), nitric acid (HNO3) (65% purity, QRëC), ammonia solution (NH3) (25% purity, Merck), hydrochloric acid (HCl) (37% purity, QRëC), thermoplastic polyurethane (TPU), dichloromethane (DCM),ultra-hard and tough (UHT) resin, HSF-1184 cell line (human skin fibroblast cell line, ATCC, Manassas, VA, USA), phosphate-buffered saline (PBS, Gibco, Grand Island, NY, USA), Dulbecco’s modified Eagle’s medium (DMEM, Gibco, Grand Island, NY, USA), fetal bovine serum (FBS, Gibco, Grand Island, NY, USA), penicillin (Gibco, Grand Island, NY, USA), streptomycin (Gibco, Grand Island, NY, USA), and trypsinase (Sigma, USA).

### 2.1. Preparation of Thermoplastic Polyurethane Solution and Maghemite Synthesis

In order to make a 3D scaffold, TPU in a solid state must be first transformed into a liquid state. So, the TPU was first dissolved with dimethylformamide (DMF). In detail, for 10% wt/v of the TPU solution, 10 g of TPU granules was dissolved with 100 mL of DMF, which was constantly stirred using a magnetic stirrer at room temperature for at least 6 h. The procedures were repeated for 12.5% wt/v and 15% wt/v of the TPU solution by increasing the amount of TPU to 12.5 g and 15 g, respectively.

In addition, the maghemite nanoparticles were synthesized based on the method described by Idris et al. [38]. Briefly, the co-precipitation method (Massart, 1981) was used to synthesize the maghemite. Ferrous and ferric chloride were added in stoichiometric amounts to an ammonium hydroxide solution by alkaline co-precipitation. Magnetite (Fe_3_O_4_), which is a black precipitate, was obtained, and then nitric acid was used to acidify the precipitate. The solution of the ferric nitrate was used to oxidize it at 100 °C to transform the solution into ϒ-Fe_2_O_3_. Then, citrate anions were used to coat the maghemite solution to prevent agglomeration between the particles. Later, the precipitate was washed with acetone and finally dispersed in water, resulting in the final stable state ϒ-Fe_2_O_3_ with a pH of 7.

### 2.2. Preparation of TPU/UHT Bio-Resin Mixed with Maghemite (ϒ-Fe_2_O_3_)

Maghemite (ϒ-Fe_2_O_3_) contains nanoparticles that act as filler, which could enhance the mechanical and biodegradability properties of the developed structure. So, in this study, three different concentrations of ϒ-Fe_2_O_3_, 1, 3, and 5% *v/v*, were added to 15% wt/v TPU solution. In order to prepare the 1% ϒ-Fe_2_O_3_ in the 15% wt/v TPU solution, 1 mL of ϒ-Fe_2_O_3_ was added to 99 mL of 15% wt/v TPU solution prepared early. This step was repeated for 3% and 5% ϒ-Fe_2_O_3_, adding 3 mL and 5 mL to the TPU solution, respectively.

### 2.3. Digital Light Processing (DLP) 3D Printing

In order to fabricate the bone scaffold, a DLP 3D printing approach was used in this study. A 3D dumbbell-shaped scaffold was designed in computer-aided design (CAD) software. The file was converted into STL format and transferred to 3D printer software named Creation Workshop. Next, the support structure was designed to ensure the connection between the product and the burn layer. Then, the sample was printed layer by layer and the TPU/UHT/ϒ-Fe_2_O_3_ was solidified as the UV light was projected onto the mixture.

### 2.4. Curing Time

It is important to determine the curing time first because the specimen must be cured perfectly. In this case, the mixed solution of 12.5% wt/v of TPU and UHT resin went through the DLP 3D printing process. Several curing times were tested in order to determine the optimum curing time for the process. The 3D structure condition was visually observed and the optimum curing time was selected when the cured body had a sharp end and precise structure.

### 2.5. Mechanical Testing

One of the major requirements of biomaterials for bone tissue engineering is the mechanical properties of the scaffold that must match the physical demand of the healthy surrounding bone tissues. Testing of the mechanical properties was conducted according to BS ISO 37:2011. In this study, the dumbbell-shaped specimen was used for the mechanical testing. The expected parameter was Young’s Modulus. The tensile test was run using an LRX Tensile Machine (Lloyd, LRX, Singapore) with a load cell of 0.5 kN at a 5 mm/min crosshead. Young’s modulus (which is a measurement of the material’s elasticity) was calculated using Equation (1);

(1) $$E_{e}=\frac{\sigma }{\varepsilon }$$

where “$E_{e}$” is Young’s modulus, “***𝜎***” is the tensile stress, and “***ε***” is the tensile strain.

### 2.6. Biodegradation Testing

A degradation test was run to evaluate the weight loss of the structure over time. Samples of the scaffold with and without ϒ-Fe_2_O_3_ were weighed at their initial weights and then immersed in a gradual beaker filled with 40 mL of simulation body fluid (SBF) at 37 °C, in accordance with the optimum body temperature. The samples were weighed weekly for a month. Next, the degraded samples were rinsed and dried in an oven at 70 °C for 5 min. Then, the dried scaffolds were weighed and recorded. Equation (2) was used to calculate the degradation rate in terms of weight loss over a month.

(2) $$\mathrm{Weight}\;\mathrm{loss}\;=\;\frac{\left(\mathrm{Initial}\;\mathrm{weight}-\mathrm{Weight}\;\mathrm{after}\right)}{\mathrm{Initial}\;\mathrm{weight}}$$

### 2.7. MTT Assay

In this study, an MTT assay was conducted to evaluate the cell proliferation rate of the human skin fibroblast cell line (HSF1184) on the TPU/UHT and TPU/UHT/ϒ-Fe_2_O_3_ 3D structures. Both the TPU/UHT and TPU/UHT/ϒ-Fe_2_O_3_ were sterilized under UV light for one hour on both sides of the structures. Then, the HSF1148 was grown in DMEM that contained 7 g/L of sodium bicarbonate (NaHCO_3_), 1% penicillin–streptomycin, and 10% fetal bovine serum (FBS). The cells were cultured at 37 °C in a humidified atmosphere containing 5% CO_2_, dissociated with 0.25% trypsin in PBS (pH 7.4), and centrifuged at 1000 r/min for 5 min at room temperature. A suitable number of normal human skin fibroblast (HSF1184) cells ($5\times 10^{4}$ cells cm^2^ seventh passage) were collected and dispersed into 20 mL of PBS, and then 200 mL of the dispersion was used for seeding the scaffold. Cell counting by the MTT assay procedure was performed after 72 h from seeding in a hemacytometer.

## 3. Results and Discussion

All the tests were performed three times, and three samples were employed for each test every week. All data are denoted as the mean ± standard deviation (SD). Significant differences were determined by performing Student’s *t* test, and a *p*-value ≥ 0.05 was indicated to be statistically significant.

### 3.1. Curing Time

Before printing the 3D scaffold structures, the exact curing times had to be determined in order to ensure every single layer of the 3D scaffold was burned by the UV laser and cured perfectly. Different materials and concentrations will have different optimum curing times. Investigations into the curing times needed to be performed for each formulation used in the study.

**Figure 2:**
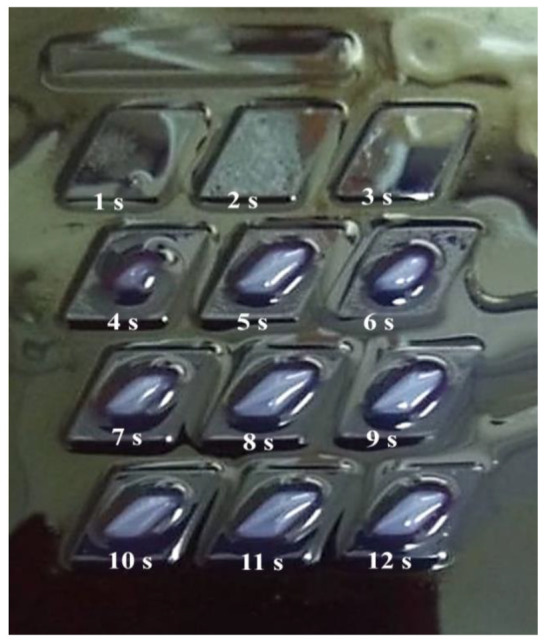
Curing times from 1 s to 12 s.

The observations and evaluations were conducted optically. In this study, the curing time was varied with three sets of experiments. For the first set, the curing time varied within 12 different periods. From the results, it was observed that the structure started to develop at 5 s and above. From 1 to 4 s, it was observed that the cured body did not develop well, as the structure was not clearly seen, as shown in Figure 2.

The experiment progressed by increasing the range of the curing time. The curing time ranged from 5 to 60 s with increments of 5 s. It was observed that the structures started to develop at 30 s and were over-cured at 40 s and above, at which point the structure was observed to melt and destroy other structures. Therefore, the structure could not be inspected at above 40 s. 

**Figure 3:**
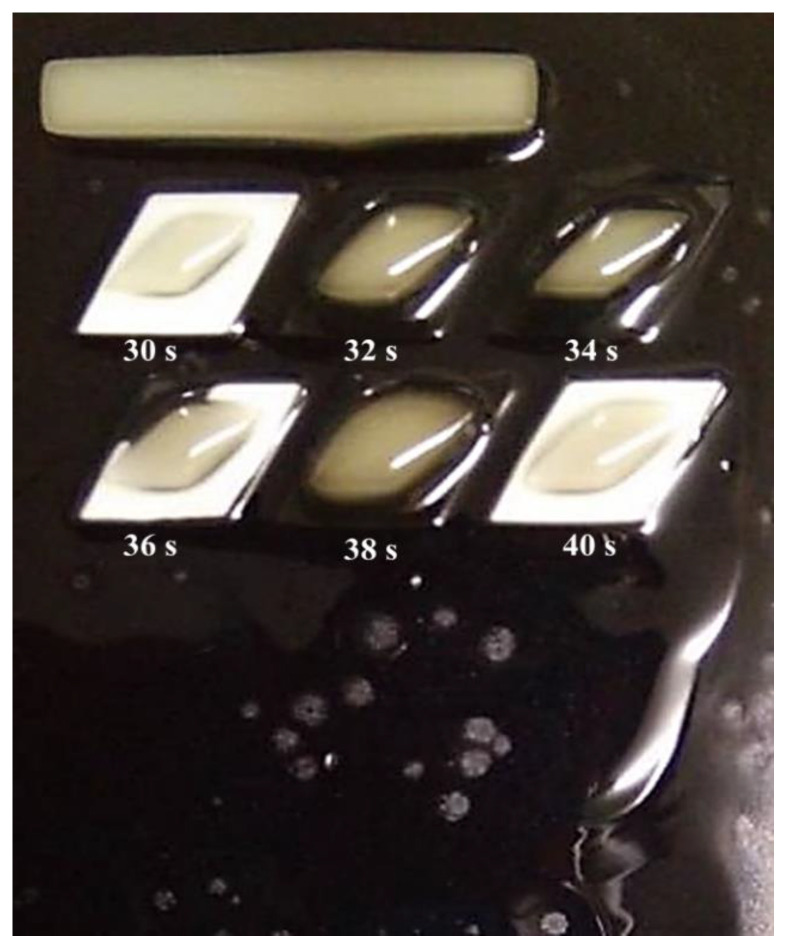
Curing times from 30 s to 40 s.

**Figure 4:**
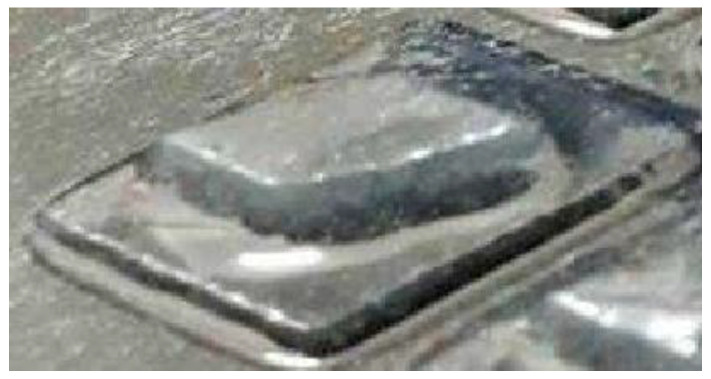
Side view of developed structure during investigation of curing times.

After knowing the specific range, the third set of experiments was conducted with the curing time starting at 30 s, with increments of 2 s, and ending at 40 s, as shown in Figure 3. From the figure, it can be observed that the best structures were cured at 30 s and 40 s. However, from a closed observation and evaluation, the cured structures at 30 s had a sharper end structure and precise structure of the inner body compared to the 40 s curing time. Thus, the optimum curing time for the TPU mixed with UHT bio-resin was 30 s. Figure 4 shows the side view of developed structure during investigation of curing times. 

### 3.2. Mechanical Properties

**Figure 5:**
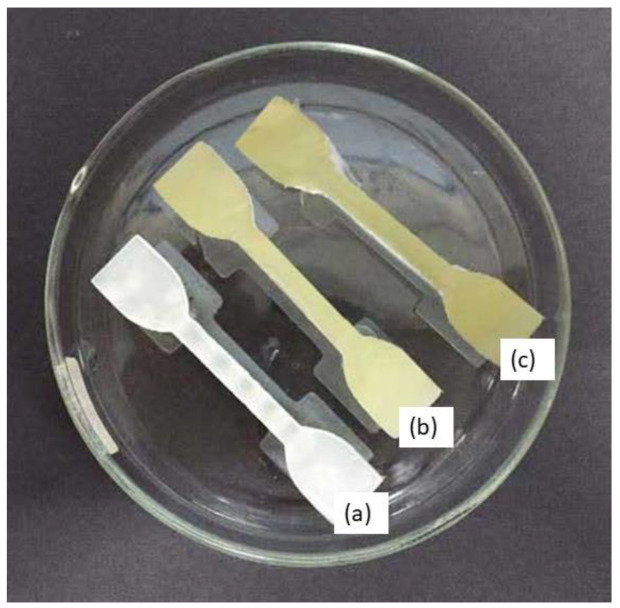
Dumbbell-shaped 3D-printed scaffold (**a**) 15% TPU + UHT; (**b**) 15% TPU+ UHT + 1% ϒ-Fe_2_O_3_; (**c**) 15% TPU+ UHT + 5% ϒ-Fe_2_O_3_.

Dumbbell-shaped scaffolds were printed using a DLP 3D printer for testing the mechanical characteristics of the specimen. Figure 5 presents the 3D scaffold printed in a dumbbell shape. The color of the dumbbell-shaped structures became more yellow and darker as the nanoparticle loading increased. 

**Figure 6:**
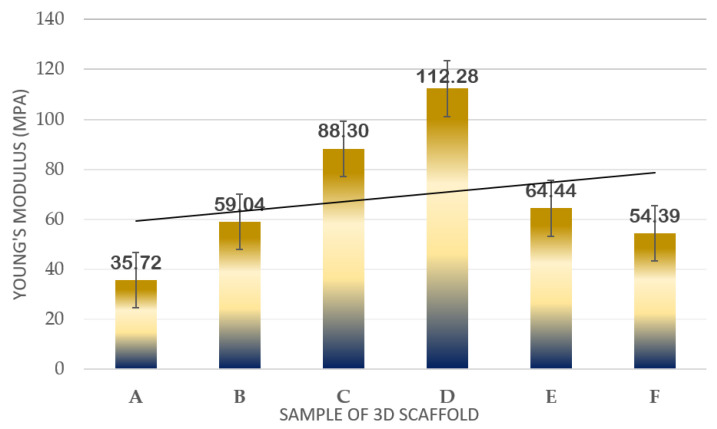
Plot of mechanical strength results in terms of Young’s Modulus of samples.

The dimensions of the printed scaffold for mechanical testing were as follows: gauge length of 30 mm, overall length of 60 mm, width of 10 mm of the narrow parallel parts, width of 20 mm at the end, and thickness of 3 mm. Table 1 and Figure 6 indicate the results of the mechanical properties in terms of Young’s Modulus for the developed 3D structure. For the first part, the mechanical test was run for TPU/UHT at three different concentrations of TPU, which were 10, 12.5, and 15% wt/v. 

In Figure 6, it can be observed that as the concentration of TPU increased, the Young’s Modulus also increased. Among the three concentrations, the highest Young’s Modulus resulted in 15% wt/v TPU/UHT with 88.30 ± 1.032 MPa due to the high strength of the covalent bond of TPU. In addition, the 15% wt/v of TPU was selected to test with three different concentrations (1, 3, and 5% v/v) of ϒ-Fe_2_O_3_ for further study. As mentioned before, ϒ-Fe_2_O_3_ acts as a filler, which enhances the mechanical properties of the structure. However, the correct concentration must be added to ensure the nanoparticles in the ϒ-Fe_2_O_3_ function well with the other solutions. Hence, in the graph, it can be observed that the highest Young’s Modulus resulted in 15% wt/v TPU/UHT with 1% ϒ-Fe_2_O_3_, which was at 112.28 ± 2.011 MPa. 

Overall, the Young’s Modulus for the three concentrations of ϒ-Fe_2_O_3_ decreased as the concentration increased. This was because the materials were disrupted with agglomerations of ϒ-Fe_2_O_3_ nanoparticles. In our previous study [33], excessive nanoparticle loading in the PVA tended to agglomerate and, thus, form cracks when spun at a very rapid speed, which subsequently contributed to a decrease in the Young’s Modulus of the electrospun nanofibrous mats. Similar findings were reported by [39], where it was reported that any additives tended to agglomerate when present in excessive amounts during mixing with other materials. An excessive number of nanoparticles in the composition was not suitable for this study.

### 3.3. Biodegradation Test

**Figure 7:**
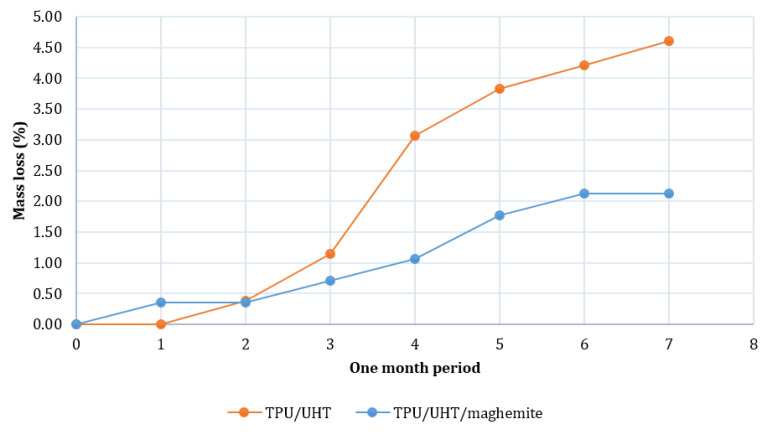
Plot of degradation rate over a period of one month.

Figure 7 shows the degradation rates of TPU/UHT and TPU/UHT/ϒ-Fe_2_O_3_ in one month. After a month, the degradation rate of TPU/UHT was 4.60%, which was higher than the TPU/UHT/ϒ-Fe_2_O_3_ structure, with a 2.12% degradation rate. For both samples, the graph shows that the weight loss increased non-linearly during the testing period. In comparison, throughout the month, the degradation rate of the TPU/UHT structure in the absence of ϒ-Fe_2_O_3_ nanoparticles was lower than the TPU/UHT structure containing ϒ-Fe_2_O_3_. This was due to the existence of magnetic nanoparticles, which made the degradation rate slower. In addition, the strong Fe-O bonds contained a polymer chain that triggered a slower degradation rate P [33]. 

### 3.4. MTT Assay

**Figure 8:**
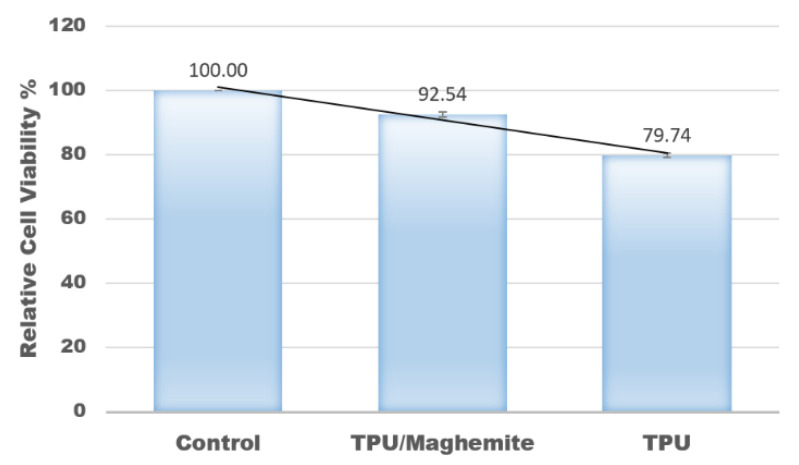
Relative values of cell viability with different composites of materials.

In this study, an MTT assay was also conducted to evaluate the cell proliferation rate of human skin fibroblast cell line (HSF1184) on the 3D structure of TPU/UHT and TPU/UHT/ϒ-Fe_2_O_3_ scaffolds. The control group had nothing except the test cells (no scaffold). Figure 8 shows the results of the relative cell viability with different structure compositions. According to the plot, the proliferation rate of the HSF1184 by TPU/ϒ-Fe_2_O_3_ was higher than the TPU structure without ϒ-Fe_2_O_3_. This was because the existence of the magnetic field in the ϒ-Fe_2_O_3_, known as the osteoinductive effect, accelerated the proliferation rate. This result is in accordance with our previous research and that of Fallahiarezoudar et al. [15]. In addition, the presence of ϒ-Fe_2_O_3_ developed a great number of tiny magnetic fields, and each nanoparticle acted as a single magnetic field that integrated with the matrix. This created a micro-environment on the surface of the blend, which made a great number of tiny magnetic fields. This situation led to an increase in the cell proliferation rate. In addition, ϒ-Fe_2_O_3_, which consists of magnetic nanoparticles has a large surface area-to-volume ratio. Thus, the existence of ϒ-Fe_2_O_3_ in TPU increased the cell area attachment, which allowed more cells to anchor [40].

## 4. Conclusions

In conclusion, the objectives were successfully achieved in this study. Firstly, the curing time for the composite material of TPU/UHT containing ϒ-Fe_2_O_3_ scaffold developed using a DLP 3D printer was 30 s. A higher concentration of TPU in the composite materials provided better mechanical strength and elastic modulus. Furthermore, the addition of ϒ-Fe_2_O_3_ enhanced the mechanical properties of the 3D structure. However, an excessive amount of ϒ-Fe_2_O_3_ could decrease the mechanical properties due to the agglomeration of the ϒ-Fe_2_O_3_ nanoparticles. The degradation rate of the TPU/UHT structure containing ϒ-Fe_2_O_3_ was lower than that of the TPU/UHT structure without ϒ-Fe_2_O_3_ due to the strong Fe-O bonds in ϒ-Fe_2_O_3_ nanoparticles. Lastly, the presence of ϒ-Fe_2_O_3_ in the structure increased the proliferation rate of HSF1148 because of numerous magnetic nanoparticles that can integrate with the cellular matrix.

## Figures and Tables

**Table 1:** Young’s Modulus of scaffolds at different concentrations with and without ϒ-Fe_2_O_3_.

Sample Label	Sample	Young’s Modulus (MPa)			
		1	2	3	Average
A	10% TPU + UHT	35.71	35.73	34.08	35.72 ± 1.001
B	12.5% TPU + UHT	59.70	57.87	60.22	59.04 ± 1.871
C	15% TPU + UHT	87.91	90.69	86.29	88.30 ± 1.032
D	15% TPU+ UHT + 1% ϒ-Fe_2_O_3_	100.65	115.13	121.08	112.28 ± 2.011
E	15% TPU+ UHT + 3% ϒ-Fe_2_O_3_	69.17	65.10	59.05	64.44 ± 2.210
F	15% TPU+ UHT + 5% ϒ-Fe_2_O_3_	54.39	52.29	55.94	54.39 ± 1.171

## Data Availability

The data presented in this study are available upon request from the corresponding author. The data are not publicly available due to the project being currently uncompleted.
